# Divergent PTEN–p53 interaction upon DNA damage in a human thyroid organoid model with germline *PTEN* mutations

**DOI:** 10.1530/ERC-24-0216

**Published:** 2025-03-08

**Authors:** Juan Andres Venegas, Omer Enes Onur, Shin Chung Kang, Masahiro Hitomi, Charis Eng

**Affiliations:** ^1^ Genomic Medicine Institute, Lerner Research Institute, Cleveland Clinic, Cleveland, Ohio, USA; ^2^ Cleveland Clinic Lerner College of Medicine, Case Western Reserve University, Cleveland, Ohio, USA; ^3^ Center for Personalized Genetic Healthcare, Medical Specialties Institute, Cleveland Clinic, Cleveland, Ohio, USA; ^4^ Taussig Cancer Institute, Cleveland Clinic Foundation, Cleveland, Ohio, USA; ^5^ Department of Genetics and Genome Sciences, Case Western Reserve University School of Medicine, Cleveland, Ohio, USA; ^6^ Case Comprehensive Cancer Center, Case Western Reserve University School of Medicine, Cleveland, Ohio, USA

**Keywords:** thyroid organoid, PTEN, p53, DNA damage, oncogenesis

## Abstract

Germline mutations in the tumor suppressor phosphatase and tensin homolog (*PTEN*) cause PTEN hamartoma tumor syndrome (PHTS). PHTS is characterized by an elevated lifetime risk of differentiated thyroid cancer (DTC), 30 times higher than the general population. However, only 1 in 3 PHTS patients develop DTC, and it remains unknown whether specific *PTEN* variants are associated with an increased risk of DTC. PTEN antagonizes the phosphatidylinositol 3-kinase (PI3K)–AKT signaling pathway, a frequently affected pathway in sporadic DTC. PTEN also acts as a guardian of the genome by interacting with other tumor suppressors. Here, we report how ionizing radiation, an environmental tumorigenic contributor, modifies the DNA damage response based on the type of germline *PTEN* variants. We hypothesized that certain *PTEN* variants associated with DTC create a pro-oncogenic molecular signature upon radiation-induced DNA damage. DTC-associated (*PTEN^M134R^*) or DTC-non-associated (*PTEN^G132D^*) germline *PTEN* mutant alleles were introduced into a human induced pluripotent cell (hiPSC) line derived from a healthy donor utilizing CRISPR-Cas9 gene editing technology. We determined radiation-induced transcriptomic changes in functional thyroid organoids induced from wild-type and both heterozygous *PTEN* mutant hiPSCs. Both bulk and single-cell RNA sequencing data indicated that radiation upregulated the p53 network more potently in the thyroid organoids with *PTEN^WT/G132D^* than those with *PTEN^WT/M134R^*, which could be mediated by AKT-dependent MDM2 inactivation and PTEN–p53 physical interaction. Our data suggest that the lack of p53 pathway activation through PTEN–p53 network interactions explains why *PTEN^M134R^* is a DTC-susceptible variant.

## Introduction

*PTEN* (phosphatase and tensin homolog) is a tumor suppressor gene whereby germline variants cause PTEN hamartoma tumor syndrome (PHTS) (Marsh *et al.* 1999, Eng *et al.* 2001, Yehia *et al.* 2023). PHTS confers a >30 times higher lifetime risk of differentiated thyroid cancer (DTC) with significantly earlier age at onset and higher predisposition to follicular subtype when compared to sporadic DTC in the general population (Ngeow *et al.* 2011, Tan *et al.* 2012, Bubien *et al.* 2013, Plamper *et al.* 2018). This evidence makes PHTS one of the main contributors of hereditary DTC (Nosé 2011). However, roughly two-thirds of patients with PHTS do not develop DTC, and very little is known about whether and how different germline *PTEN* variants interact with disease inducers, such as environmental radiation, and how they interact with other tumor suppressor networks to prevent or promote DTC onset.

PTEN’s canonical function is to antagonize the phosphatidylinositol 3-kinase (PI3K)–AKT signaling pathway, one of the most affected pathways in sporadic DTC (Myers *et al.* 1997, Agrawal *et al.* 2014). PTEN also plays a role as a guardian of the genome by promoting DNA repair, cell cycle arrest, and chromosomal stability, among other functions (Kandel *et al.* 2002, Shen *et al.* 2007, Planchon *et al.* 2008, Bassi *et al.* 2013, Hitomi *et al.* 2023). PTEN does so through canonical and non-canonical means, including the interaction between PTEN and p53 molecular signaling networks. On the one hand, PTEN promotes p53 function in cancer cells by inhibiting MDM2 function in a lipid phosphatase-dependent manner (Freeman *et al.* 2003). On the other hand, PTEN–p53 physical interaction prevents p53 degradation by MDM2 (Stambolic *et al.* 2001, Tang & Eng 2006). In addition, p53 can transcriptionally regulate PTEN by binding to the *PTEN* promoter (Mayo *et al.* 2002). More recently, a combination of bioinformatic approaches and subsequent validation in cancer cell lines has shown that PTEN and p53 networks act in concert to determine the type of cellular response during DNA damage repair (DDR) (Gupta *et al.* 2023). So, exploring the *PTEN* genotype-specific alteration of the complex relationship between these two molecular networks to influence DDR may be pertinent to understanding why some PHTS patients develop DTC while others do not, particularly because p53 activation is a critical response to radiation, a major environmental etiology of DTC.

Children who have undergone radiological splenectomy therapy have roughly twice the risk of developing DTC than matched controls (de Vathaire *et al.* 2015). Furthermore, the increase in DTC incidence after radiation exposure has been very well documented, a causal effect has been established, and children and adolescents appear to be the most affected population (Ron *et al.* 1995, Sinnott *et al.* 2010, Hong *et al.* 2019, Drozd *et al.* 2021). As relevant to PHTS, DTC is one of the most prevalent component cancers in this syndrome and is the cancer with the youngest ages at diagnosis (Ngeow *et al.* 2011, Plamper *et al.* 2018). As such, we sought to investigate whether the PTEN and p53 interaction during radiation-induced DDR in the context of PHTS may be associated with DTC.

In this work, we generated five human induced pluripotent stem cell (hiPSC) lines harboring each of two distinct mutant *PTEN* alleles, *M134R* associating with DTC and *G132D* not associating with DTC in hetero- or homozygous states by using CRISPR-Cas9 gene editing. Upon irradiation of thyroid organoids derived from these iPSC lines, we observed a divergent regulation of the p53 pathway between the two mutant genotypes. *PTEN^WT/G132D^* (*WT/G132D)* thyroid organoid upregulates the p53 pathway when compared to *PTEN^WT/M134R^* (*WT/M134R)* and *PTEN^WT/WT^* (*WT/WT*) thyroid organoids. This phenomenon may have a protective effect on the thyroid tissue of patients with *WT/G132D* genotype when compared to patients with *WT/M134R* genotype, suggesting that this type of approach could potentially help define individualized genotype-environment x phenotype correlations in PHTS.

## Materials and methods

### Human induced pluripotent stem cell (hiPSC) culture

Using CRISPR/Cas9 technology, two distinct *PTEN* variants, PC640. PTEN. g4 132G>D and PC640. PTEN. g7 134M>R, were introduced into a commercially available healthy male skin fibroblast-derived hiPSC line (BJFF.6) by the Washington University Genome Engineering & Stem Cell Center, iPSC Core (GEiC, Washington University School of Medicine, USA) (Chen & Pruett-Miller 2018). The zygosity of the introduced variants was confirmed by next-generation sequencing, and two or three different clones were expanded for each PTEN-mutant and the PTEN-WT line. These established *PTEN*-mutant hiPSC lines and parental lines were maintained in a supplemented StemFlex™ basal medium (ThermoFisher Scientific, USA; cat. no. A3349301) in plates that were pre-coated with 1.6 μL/mL Geltrex™ LDEV-Free Reduced Growth Factor (Gibco™, ThermoFisher Scientific, USA; cat. no. A1413202) in DMEM (Gibco™ DMEM, ThermoFisher Scientific; cat. no. 11995065) for overnight incubation at 37°C.

### Cell viability assay and doubling time calculation

hiPSCs were plated at ∼50% confluency in 96-well plates. The alamarBlue™ Cell Viability Reagent (ThermoFisher; cat. no. DAL1025) was used according to the manufacturer’s protocol. Following an established protocol (Dinh *et al.* 2024), the same plate was read at 0 h (one day after plating the cells), 24, 48, and 96 h at 600 nm wavelength with a BioTek Synergy H1 Multimode Reader (Agilent, USA; cat. no. 7191000). Optical density values were normalized to 0 h = 1 for each genotype. Following data acquisition, the doubling time was calculated by applying the following equation to the data only during the exponential *phase of growth (0–24 h): $\text{doubling}\;\text{time}=\frac{\text{duration}*\log\left(2\right)}{\log\left(\text{final}\;\text{concentration}\right)-\log\left(\text{initial}\;\text{concentration}\right)}$

### Scratch-wound healing assay

hiPSCs were cultured until they reached 90–100% confluency and a straight scratch was made using a 1-mL pipette tip. Images were taken using an EVOS XL Core Imaging System (ThermoFisher; cat. no. AMEX1000), and every field was marked with a square. Images were taken 24 h later at the same location utilizing the marked fields, and ImageJ (FIJI, GitHub, version 2.0.0-rc-69/1.52n) was calibrated for distance scale with the picture of a Neubauer chamber inscribed with lines with defined intervals that were taken with the same imaging setup using the same objective lens.

### Induction of thyroid organoids in 3D culture

Thyroid organoids were generated from feeder-free hiPSC cultures using a previously reported protocol (Kurmann *et al.* 2015) with minor modifications, as follows: One day prior to differentiation induction (day 0), embryoid bodies (EBs) were generated from hiPSC clusters grown as monolayers, which were detached from the plate via Collagenase IV (MilliporeSigma, USA; cat. no. 17104019) digestion for 45- to 90-min incubation at 37°C in a humidified atmosphere with 5% CO_2_. Detached clusters of iPSCs were collected into a 15-mL centrifuge tube with a 5-mL serological pipette and sedimented by gravity and resuspended in the StemFlex™ medium. The clusters were incubated for 24 h in 6-well plates. On day 0, the StemFlex™ medium was discarded and EBs were washed with phosphate-buffered saline (PBS), allowing them to sediment to the bottom of the tube by gravity, and an endoderm induction kit (ThermoFisher Scientific; cat. no. 05111) medium was added. EBs were placed in 6-well plates and cultured with continuous shaking (ThermoFisher Scientific; cat. no. 88881101) at 80 revolutions per minute and incubated at 37°C and 5% CO_2_. As described in Kurmann *et al.* (2015), on day 4, anterior endoderm medium was added (briefly, complete serum-free differentiation medium (cSFDM) consisting of 75% IMDM (Invitrogen, USA; 12440) and 25% Ham’s modified F12 medium (Cellgro; 10-080-CV) supplemented with N2 and B27+RA (Invitrogen; 17502-048 and 17504-44), 0.05% bovine serum albumin (BSA) (Invitrogen; 15260-037), 200 mM L-glutamine (Invitrogen; 25030-081), 0.05 mg/mL ascorbic acid (Sigma, USA; A4544) and 4.5 × 10^−4^ M monothioglycerol (MTG) (Sigma; M6145) was supplemented with 2 μM dorsomorphin (Stemgent, USA; 04-0024) and 10 μM of SB431542 (Tocris; 1614)). On day 9, the medium was switched to a thyroid precursor induction medium, which was changed every day (cSFDM supplemented with 10–100 ng/mL rhBMP4 (R&D; 314-BP), 100–250 ng/mL rhFGF2 (R&D; 233-FB) and 100 ng/mL heparin salt (Sigma; H4784)). Finally, on day 25, the medium was switched to a thyroid maturation medium (Ham’s F12 supplemented with 15 mM HEPES, 0.8 mM CaCl_2_, 100 ng/mL heparin sodium salt, 0.25% BSA, 50 ng/mL mIGF-1 (R&D; 791-MG), 5 μg/mL insulin (Millipore; I9278), 5 μg/mL ITS (Corning, USA; 354352), 25 ng/mL hEGF (R&D; 263-EG), 50 nM dexamethasone (Sigma; D4902) and 100 mU/mL hTSH (Millipore; 869006)). Thyroid organoids were maintained in the maturation medium until around day 35.

### Immunofluorescence and imaging

Thyroid organoids were fixed for 15 min at 4°C with 4% paraformaldehyde in PBS at a pH of 7.4. Fixed organoids were washed with PBS and incubated overnight at 4°C with 30% sucrose dissolved in PBS prior to embedding with Tissue-Tek O.C.T. Compound (Sakura Finetek, USA; cat. no. 4583) using Tissue-Tek Cryomold (Sakura Finetek;. cat. no. 4565). Embedded organoids were cryo-sectioned at 11 μm thickness using a cryostat (Leica; CM 1850 Cryostat) and affixed to polarized glass slides (ThermoFisher Scientific, Fisherbrand; cat. no. 12-550-15). Tissues were permeabilized and blocked for 1 h in 0.5% Triton X-100 and 10% normal goat serum (Vector Laboratories, USA; cat. no. S-1000) in PBS at room temperature. After blocking, tissues were incubated overnight at 4°C with primary antibodies in 10% normal goat serum in 0.5% Triton X-100 in PBS. After washing three times with PBS for 30 min at room temperature, tissues were incubated for 2 h at room temperature with secondary antibodies diluted in 0.5% Triton X-100 in PBS. Sections were then washed three times for 15 min each with PBS, and then, slides were mounted on slides with anti-fade mounting buffer containing 4′,6-diamidino-2-phenylindole (DAPI) (Vector Laboratories; cat. no. H-1200). Images from immunostaining were acquired using the Leica TCS-SP8-AOBS inverted confocal microscope (Leica; SP8 confocal microscope). The total number of cells in each field was determined by counting DAPI-stained nuclei. Fluorescence intensity was calculated by creating a watershed mask based on DAPI-stained nuclei to separate each individual nucleus and calculate the mean fluorescence intensity of each of them using ImageJ (FIJI, GitHub, version 2.0.0-rc-69/1.52n). At a minimum, three fields were analyzed for each sample, and all experiments were repeated at least three independent times.

### Colocalization analysis

Cells were dissociated from thyroid organoids by incubation with TrypLE™ (ThermoFisher Scientific, Fisherbrand; cat. no. 12563-011) and were plated onto Geltrex-coated coverslips to form 2D cultures. Immunofluorescence was performed as stated above, and z-stacks of the slides were taken with a distance between stacks of 0.25 μm (20–30 stacks per slide). 3D images were reconstructed using the Volocity® 3D Image Analysis software (Quorum Technologies, UK). Once the 3D image was reconstructed, thresholds were set (Costes *et al.* 2004) and the colocalization analysis was performed. Overlap coefficients were used to calculate the colocalization between the green and red signals.

### Western blot and co-immunoprecipitation analysis

Organoids were collected into microcentrifuge tubes by gravity sedimentation and washed three times with PBS, and ∼20 organoids were lysed for 1 h on ice with M-PER™ (Mammalian Protein Extraction Reagent) buffer (ThermoFisher Scientific; cat. no. 78505) supplemented with phosphatase and protease inhibitor cocktails (MilliporeSigma; cat. no. P5726, P0044, P8340). Supernatants were collected after centrifugation at 4°C for 15 min at 16,000 *g*, and protein concentration was determined by bicinchoninic acid (BCA) assay according to manufacturer’s directions using BCA™ Protein Assay Kit (ThermoFisher Scientific; cat. no. 23228, 1859078). A total of 20–50 μg of protein per sample was loaded onto 4–15% polyacrylamide Criterion gels (Bio-Rad, USA; cat. no. 64496731). Separated proteins were electrotransferred to a nitrocellulose membrane using a Bio-Rad Turbo Blot system. The blots were blocked with 10% BSA in Tris-buffered saline, pH 7.4, with 0.025% Tween-20 (TBST), incubated for 1 h at room temperature prior to overnight incubation with the primary antibody in the blocking buffer at 4°C. The next day, after washing three times for 5 min with TBST, the membrane was incubated with either horse radish peroxidase-conjugated secondary anti-mouse antibody (Promega, USA; cat. no. PRW4021) or anti-rabbit antibody (Promega; cat. no. 4011) for 1 h at room temperature. Blots were imaged using a ChemiDoc MP Imaging System (Bio-Rad; cat. no. 12003154), to capture immunoreactive bands that were visualized by incubating with ECL reagent SuperSignal™ West Pico PLUS Chemiluminescent Substrate (ThermoFisher Scientific; cat. no. 34578). All primary antibodies were diluted to 1:1,000 and 1:2,000 for secondary antibodies. Band intensity analysis was performed using ImageJ (software version 2.0.0-rc-69/1.52n). For co-immunoprecipitation, cell lysate containing 75–100 μg protein at a concentration of 1 μg/μL was used per precipitation. The lysate received 10 μL of magnetic beads (ThermoFisher Scientific; cat. no. 10003D), which were previously washed (with M-PER™) and loaded with the antibody of choice (2 µg of antibody for each 10 μL of beads) by 2-h incubation followed by M-PER wash. The resulting mixtures were incubated with continuous shaking at 4°C overnight. On the next day, the supernatant was collected after the beads were sedimented by centrifugation. The beads fraction was washed and resuspended in TBST buffer with phosphatase and protease inhibitor cocktails. After boiling with the sample buffer, the precipitated proteins released from the beads were analyzed by western blotting as described above (antibody list in Supplementary Table 1, see section on supplementary materials given at the end of this article).

### ELISA for T4 quantification

Media of mature thyroid organoid cultures were collected on day 35. Human Free Thyroxine (fT4) ELISA Kit (MyBioSource, USA; cat. no. MBS266723) was used following the manufacturer’s protocol. ELISA signals were determined by measuring absorbance at 450 nm using a BioTek Synergy H1 Multimode Reader (Agilent; cat. no. 7191000). T4 concentrations in the samples were calculated using a reference curve that was plotted with the T4 standard supplied in the kit.

### Irradiation of thyroid organoids and cells

To induce DNA damage, cells or organoids were exposed to indicated doses of ionizing irradiation using the Shepherd ^137^Cs Irradiator (JL Shepherd & Associate, USA). Following a dose of 3 Gy, the organoids/cells were placed back in the incubator, and after the appropriate times, they were harvested for cell lysate preparation or fixed for immunofluorescence.

### Cell dissociation from organoids for single-cell RNA sequencing

Fifteen to twenty thyroid organoids with or without 3 Gy exposure were transferred to a 22.1 cm^2^ round tissue culture dish 8 h after exposure (TPP Techno Plastic Products, Switzerland; cat. no. 93060) and washed with PBS three times. Organoid digestion was carried out using the TrypLE™ Select Enzyme (Gibco™, ThermoFisher Scientific; cat. no. A1217701). 3 mL TrypLE™ Select Enzyme were added to each well, and the samples were incubated with shaking (70 rpm) at 37°C and 5% CO_2_ for 15 min. 9 mL cold 10% BSA in HAM-12 media were added to each well to stop the digestion, and the digested organoids were triturated by pipetting. The dissociated cells were transferred to the 15-mL conical tube and centrifuged at 1,000 rpm for 3 min at room temperature. After resuspension in an appropriate volume of PBS, the cells were then passed through a 40-μm Corning© Cell Strainer (Corning, ThermoFisher Scientific; cat. no. 431750). Cells were counted with trypan blue to assess cell viability. Cell concentration was adjusted to 1,000 cells/μL, and samples showing >80% viability were used for sequencing.

### Sample processing with 10x Genomics and cDNA library preparation

Using 10x Genomics Chromium Single Cell 3′ Reagent Kits (version 3.1), 6,000 to 10,000 cells per sample were captured for library and sequencing preparation. Following the organoid dissociation, the single-cell suspension, the gel beads, and the emulsion oil were added to the 10x Genomics Single Cell Chip G. Immediately following the droplet generation, samples were transferred to a PCR 8-tube strip (USA Scientific) for reverse transcription reaction using a SimpliAmp thermal cycler (Applied Biosystems, USA). cDNA generated by reverse transcription was recovered using the recovery reagent provided by 10x Genomics. The cDNA was cleaned up using the Silane DynaBeads according to the 10x Genomics user guide. The purified cDNA was amplified for 11 cycles and subsequently cleaned up using SPRIselect beads (Beckman Coulter, USA). To determine the cDNA concentrations, 1:10 dilution of each sample was analyzed on an Agilent Bioanalyzer High Sensitivity chip. The cDNA libraries were constructed according to the Chromium Single Cell 3′ Reagent Kit version 3.1 user guide.

### scRNA-seq data analysis

The scRNA-seq libraries were sequenced to a target depth of 50,000 read pairs per cell. De-multiplexed FASTQ files were processed using cellranger-4.0.0. Reads were mapped using the count pipeline. Reference genome refdata-gex-GRCh38-2020A and GTF from GENECODE v32 (GRCh28.p13) were used to align the reads. Filtering, normalization, and downstream analysis were performed using Seurat 3.2.1 in R (Hao *et al.* 2021). Briefly, the raw data were filtered for low-quality cells using the following QC thresholds. Cells with mitochondrial reads >25% of total mapped reads, gene counts <250 or gene features >40,000 were filtered, leaving 47,864 high-quality cells for the downstream analysis. Seurat’s standard workflow was followed. Using the 2,000 most variable genes, principal components (PCs) were computed, and the first 15 PCs were utilized to generate clustering at resolution 0.45 and UMAP was generated.

### Cell cycle scoring

Two cell scores were computed for each barcode in the sample. Cell cycle states were imputed to be in S, G1, or G2/M based on these two scores using the Seurat cell score tool. Results were plotted in a UMAP, and a bar graph was generated to depict the percentage of cells in each cell cycle state.

### p53 inhibition and bulk RNA-seq insolation, sequencing, and processing

For p53 inhibition treatment, cells were exposed to 15 μM pifithrin-α hydrobromide (Tocris; cat. no. 63208-82-2) for 24 h and irradiated as described previously, and the treatment was maintained after the irradiation. A standardized kit for RNA isolation was used following manufacturer’s instructions in pre- and post-irradiated organoids (QIAGEN, USA; cat. no. 74104). The total RNA libraries were prepared following the manufacturer’s protocol (Illumina, USA; Document #1000000124514). In summary, ribosomal RNA was depleted, and the RNA underwent fragmentation and denaturation. Subsequently, cDNA was synthesized, followed by end repair, adenylation and adaptor ligation, culminating in PCR amplification. Finally, the libraries were evaluated for quality, quantified and sequenced on an Illumina platform (NovaSeq 6000). Low-quality reads and Illumina adapter sequences were trimmed using fastp version 0.21.0 (Chen *et al.* 2018). STAR version 2.7.10.a (Dobin *et al.* 2013) was used to map the trimmed reads to the GRCh38 reference genome to generate a gene count matrix with parameter ‘—quantMode’. Differential expression analyses were conducted using DESeq2 version 1.40.2 (Love *et al.* 2014).

### Transcriptomic profile comparison and pathway enrichment analysis

Differential expression analyses were conducted between previously filtered and normalized samples using the Loupe Browser tool (10x Genomics) and log2fold parameters.

The transcriptome of each sample was examined against that of the entire (all samples combined) data set as well as against each one individually. Only the cells with high-count (approximately 600 genes) were considered as data sets to be used for ingenuity pathway analysis (IPA, QIAGEN). Only those differentially expressed genes (DEGs) with log2fold changes >1 or <−1 and *P*-value <0.05 were selected for standard downstream core analysis using IPA. Enrichment for diseases and functions was also examined using IPA.

### Flow cytometry quantification of γ-H2AX

Irradiated and non-irradiated thyroid organoids were dissociated to prepare single cell suspensions as previously described. Cells were washed with PBS three times and DAPI was added as a live/dead marker using a concentration of 0.1 µg/mL in PBS for 20 min, after which stained cells were washed three times and left on ice. A commercial kit was used to fixate and permeabilize the cells following the manufacturer’s instructions (BioLegend, USA; cat. no. 424401). γ-H2AX intensities were quantified using a conjugated primary antibody for two hours (BioLegend, cat. no. 613411). Cells were tested in a 5-laser BD LSRFortessa II (BD Biosciences, USA), and data were analyzed using FlowJo v10.10 (Becton Dickinson).

### Statistics

Data were expressed as mean ± SD (standard deviation) unless otherwise described. Student’s *t*-test or one-way ANOVA (with Tukey’s post hoc test) was performed when appropriate, to compare between or among groups, respectively. All Student’s *t*-tests were two-tailed and unpaired. We considered all *P*-values below 0.05 statistically significant. Statistical analyses were performed in GraphPad Prism 8, Loupe Browser 6, or IPA.

### Data availability

Raw data for this study were generated at Cleveland Clinic’s Genomics Core. Derived data supporting the findings of this study are available from the corresponding author upon request.

### Code availability

The present study utilized previously published approaches, of which codes are shared on public repositories: Seurat (https://github.com/satijalab/seurat), cellranger (https://github.com/10Xgenomics/cellranger), Loupe Browser (https://github.com/10Xgenomics/loupeR), fastp (https://github.com/OpenGene/fastp), STAR (https://github.com/alexdobin/STAR/releases) and DESeq2 (https://bioconductor.org/packages/release/bioc/html/DESeq2.html).

## Results

### Increased cell proliferation and migration in the *PTEN^WT/G134D^* hiPSC

**Figure 1:**
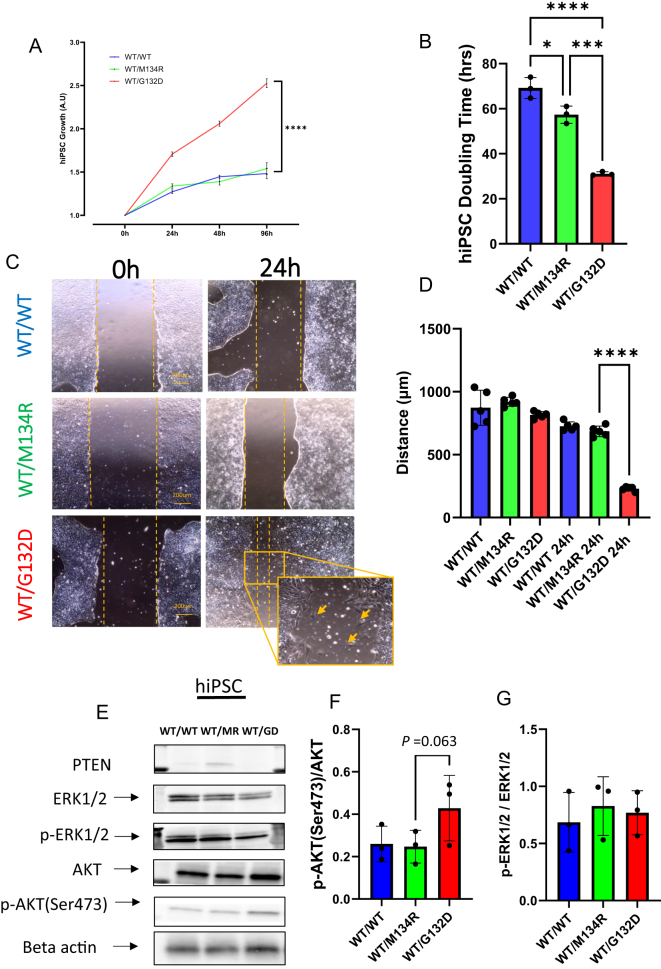
*PTEN^WT/G132D^* increases cell proliferation and migration when compared to *PTEN^WT/M134R^*. (a) Growth curve of hiPSCs with the three PTEN genotypes. Viability was assessed at 0 h (one day after seeding), 24, 48 and 96 h; *F* = 1,011; *P*-value = <0.0001. (b) Doubling time of hiPSCs with the three different PTEN genotypes; *F* = 92.43; adjusted *P*-value: * = 0.0143, *** = 0.0002, **** = <0.0001. (c) Representative bright-field images of the wound healing assay, 0 and 24 h time points in the three PTEN genotypes. The yellow arrows point to cells detached from the main cell cluster. (d) Quantification of the wound healing assay measuring the distance between the borders of the scratch; *F* = 183; adjusted *P*-value = <0.0001. (e) Representative western blots of PTEN, ERK1/2, p-ERK1/2, AKT, and p-AKT(S473). (f) Western blot quantification of p-AKT(Ser473)/AKT ratio; *P*-value = 0.063. (g) Western blot quantification of p-ERK1/2/ERK1/2 ratio.

We first characterized the cellular phenotype of hiPSCs with the following *PTEN* genotypes: *WT/WT*, *WT/M134R,* and *WT/G132D*. *WT/G132D* hiPSCs exhibited a significant increase in cell viability after 96 h of seeding when compared to the *WT/WT* and *WT/M134R* cells and never reached a plateau in the growth curve within the observed duration (Fig. 1A). When we calculated the doubling time during the first 24 h, the period when both *WT/WT* and *WT/M134R* cells displayed the most rapid growth rates, we found *WT/G132D* cells proliferated ∼two-fold faster than the other two genotypes (Fig. 1B). Next, we interrogated cell migration using a scratch-wound healing assay, measuring the distance filled by the migrating cells over time by comparing the cell-free space between 0 and 24 h after scraping. The *WT/G132D* cells displayed significantly faster migration than the *WT/M134R* or *WT/WT* cells (Fig. 1C and D). Interestingly, we observed that the *WT/G132D* cells also displayed some migrating hiPSCs without cell-to-cell contacts (yellow arrows in Fig. 1C). This phenomenon is uncommon in hiPSCs since part of their stem cell molecular program maintenance depends on cell-to-cell contacts (Oyamada *et al.* 2013, Beckmann *et al.* 2016). It also suggested that, in the pluripotent state, the *WT/G132D* cells may activate some epithelial–mesenchymal transition (EMT) signaling pathways when compared to the *WT/M134R* or *WT/WT* cells. Puzzled by these observations, we evaluated PTEN downstream signaling by western blot analyses of the phosphorylation states of the kinases canonically regulated by PTEN dual phosphatase activity (Myers *et al.* 1997) (Fig. 1E). We observed a trend (*P* = 0.063) of increased p-(Ser473) AKT/AKT ratio in the *WT/G132D* cells compared to the *WT/WT* or *WT/M134R* cells (Fig. 1F). However, the p-ERK1/2/ERK1/2 ratio was not affected by the difference in genotype (Fig. 1G). These findings suggest that the *WT/G132D* hiPSCs possibly have a decreased lipid phosphatase activity without changes in the protein phosphatase activity. In addition to AKT activation, we also speculate that there are non-canonical functions of PTEN linked to these cellular and molecular observations.

### Functional thyroid organoid differentiation in all three tested *PTEN* genotypes *PTEN^WT/WT^*, *PTEN^WT/G132D^* and *PTEN^WT/M134R^*

**Figure 2:**
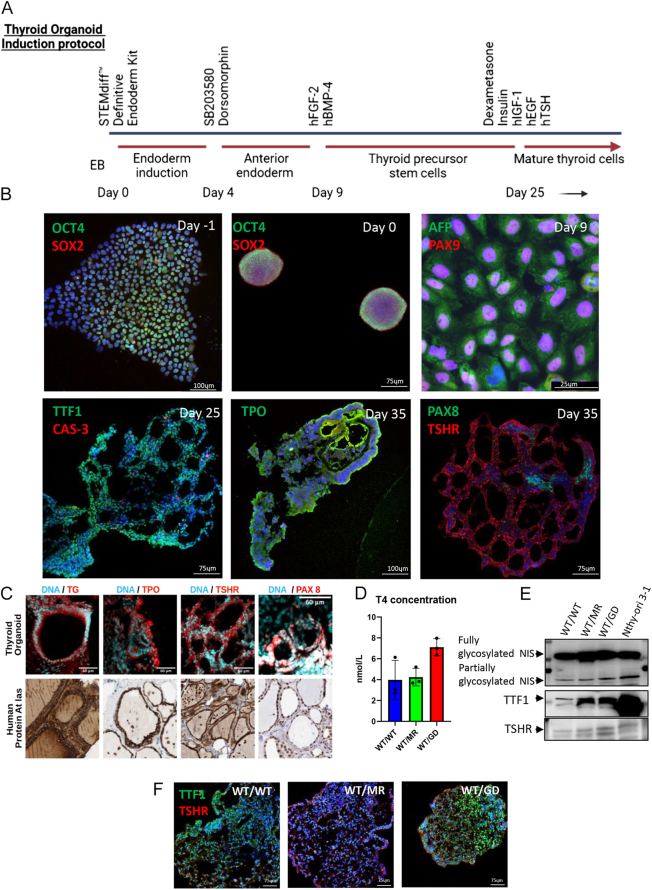
Human thyroid organoids resemble human thyroid gland morphology and function. (a) Schematic of thyroid organoid induction protocol. (b) Immunofluorescence (IF) images showing day −1 hiPSCs expressing OCT4 and SOX2, day 0 EBs positive for OCT4 and SOX2, day 9 AFP+ and PAX9+ anterior endoderm cells, day 25 thyroid precursor organoid positive for TTF1 with limited expression of cell death marker-cleaved CAS3 and day 35 mature thyroid organoids that express TPO, PAX8 and TSHR. (c) A panel of IF images capturing TG, TPO, TSHR, and PAX8 expressions in wild-type *PTEN* thyroid organoids that show a resemblance of subcellular localization (nuclear vs cytoplasm) and follicular morphology when compared to the immunohistochemistry images of normal human thyroid gland taken from the Human Protein Atlas. (d) ELISA-detected thyroid hormone (T4) in the lysates from the thyroid organoid of the three PTEN genotypes (kit sensitivity = 0.6 nmol/L). (e) Western blots of NIS, TTF1, and TSHR in the three genotypes and the human thyroid cell line S-THY 1-3, a positive control. (f) IF panel showing *WT/WT*, *WT/M134R,* and *WT/G132D* mature thyroid organoids positive for TTF1 and TSHR.

We directed hiPSCs to differentiate into thyroid organoid states by replicating multiple differentiation-inducing stimuli that drive embryo cells to carry out processes of organogenesis using recombinant human proteins (Fig. 2A). We used immunofluorescence techniques to assess how the expression of the key developmental genes was induced during the various phases of the protocol. The main goal was to detect the expression of thyroid mature markers such as NIS (sodium iodine importer), TTF1 (thyroid transcription factor 1), and TSHR (thyroid-stimulating hormone receptor). We started with a pluripotent stem cell state in 2D culture, and at day −1, cells were dislodged from the plate by collagenase treatment to form 3D EBs (OCT4+/SOX2+) at day 0. On day 9, we could detect AFP+ (pan-endoderm marker) cells and PAX9+ (anterior endoderm marker) cells. Later, on day 25, organoids contained the expression of TTF1+ cells (an early marker of primitive thyroid tissue). Finally, on day 35, the organoid cells exhibited mature thyroid markers (TPO, PAX8, and TSHR) and resembled human thyroid morphology by showing follicular structures (Fig. 2B, Supplementary Fig. 1). We then compared the localization and polarization of mature thyroid markers in organoids to those in the human thyroid gland using images from the Human Protein Atlas (Thul *et al.* 2017), finding a resemblance between the two data sets (Fig. 2C). We then assessed the function of the thyroid organoid tissue by measuring T4, the production of which requires the functionality of the whole follicular structure. We detected T4 in the thyroid organoid lysate of the three *PTEN* genotypes by ELISA (Fig. 2D). We then compared the expression of key thyroid markers between the three organoid lysates and an immortalized human normal thyroid cell line (Nthy-ori 3-1). We found that thyroid organoids with each different genotype expressed NIS, TTF1 and TSHR as our positive control (Nthy-ori 3-1) (Fig. 2E, Supplementary Fig. 1). Finally, we showed that hiPSC-derived thyroid organoids with each of the three genotypes express TTF1 and TSHR by immunofluorescence (Fig. 2F). Overall, these results demonstrated that the organoids derived by the differentiation protocol faithfully recapitulated the thyroid developmental processes and gain thyroid function *in vitro* regardless of *PTEN* status.

### p53 activation defect after irradiation in thyroid organoids with the *PTEN^WT/M134R^* genotype

**Figure 3:**
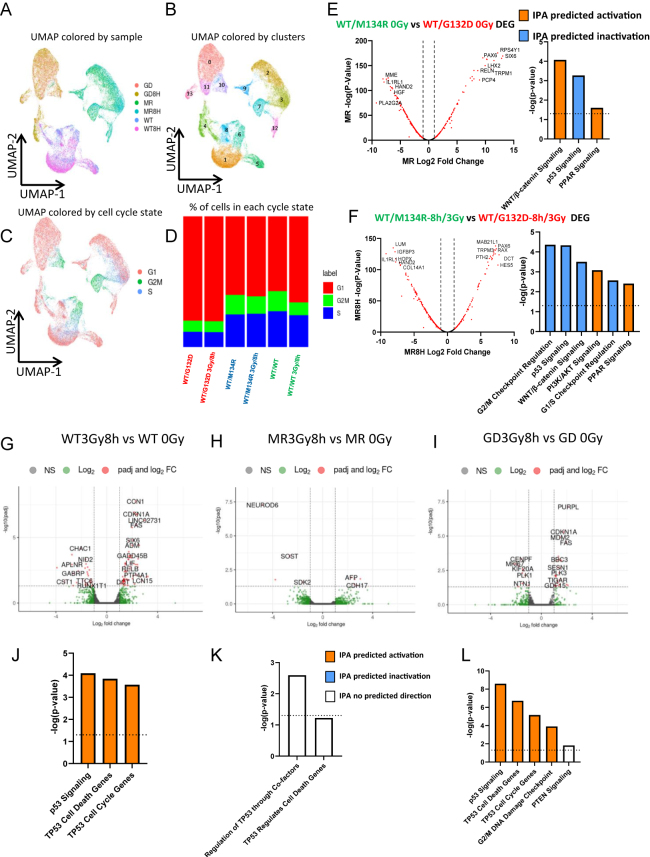
scRNA-seq and pseudo-bRNA-seq analyses of DEG predict higher G_1_ cells and p53 signaling activity in *PTEN^WT/G132D^*. (a) scRNA-seq UMAP labeled by sample type: *WT/G132D* (GD), *WT/G132D* 8 h after 3 Gy dose (GD8H), *WT/M134R* (MR), *WT/M134R* 8 h after 3 Gy dose (MR8H), *WT/WT* (WT) and *WT/WT* 8 h after 3 Gy dose (WT8H). (b) scRNA-seq UMAP labeled by k-means clusters 1–12. (c) scRNA-seq UMAP labeled by cell cycle phases G1, G2/M, and S. (d) Bar graph showing the proportion of each cell cycle phase in each sample. (e) IPA of up-/downregulated pathways in *WT/M134R* 0 Gy vs *WT/G132D* 0 Gy organoids. (f) IPA of up-/downregulated pathways in *WT/M134R*-8 h/3 Gy vs *WT/G132D-*8 h/3 Gy organoids. (g) Volcano plot up-/downregulated transcripts in *WT/WT-*8 h/3 Gy vs *WT/WT* 0 Gy organoids. (h) Volcano plot up-/downregulated transcripts in *WT/MR-*8 h/3 Gy vs *WT/MR* 0 Gy organoids. (i) Volcano plot up-/downregulated transcripts in *WT/GD-*8 h/3 Gy vs *WT/GD* 0 Gy organoids. (j) IPA predictions of up-/downregulated pathways in *WT/WT*-8 h/3 Gy organoids vs *WT/WT* 0 Gy organoids. (k) IPA predictions of up-/downregulated pathways in *WT/MR*-8 h/3 Gy organoids vs *WT/MR* 0 Gy organoids. (l) IPA predictions of up-/downregulated pathways in *WT/GD*-8 h/3 Gy organoids vs *WT/GD* 0 Gy organoids.

To assess the impact of *PTEN* germline variants on the DNA damage response of thyroid organoids, we analyzed γ-irradiation-induced transcriptomic changes using single-cell RNA sequencing (scRNA-seq) using thyroid organoid cells that were collected before and at 8 h after a 3 Gray (Gy) dose of gamma radiation (previously established dose, Supplementary Fig. 2). An initial quality control of the data set yielded a total of 47.864 high-quality beads. We performed nonlinear dimensional reduction (UMAP) of the data and categorized the cell populations using the k-means clustering strategy. We assessed the expression of thyroid-specific transcripts such as *TTF1*, *TTF2*, *PAX8*, *TSHR*, *SLC5A5*, *TG,* and *TPO*, but found very low read counts in an apparently random distribution in the UMAP, making it difficult to assess the different cell types in our data set (Supplementary Fig. 4a). We found that the data set mainly clustered by the *PTEN* genotype (Fig. 3A and B), so we decided to run a DEG analysis based on the different genotypes. DEG and subsequent IPA done on only the high count genes (approximately 600 genes) predicted that the p53 pathway was differentially regulated by γ-irradiation for the *PTEN* mutant genotypes. When we compared the heterozygous mutant genotypes against each other, IPA predicted a lack of activity of the p53 signaling pathway in *WT/M134R* organoids compared to that of *WT/G132D* organoids under both pre- and post-irradiation conditions (Fig. 3E and F). This phenomenon was also shown in a global comparison analysis of each sample and by filtering the p53+ subset (Supplementary Fig. 3 and Supplementary Fig. 5).

Since cell cycle checkpoints are activated in response to DNA damage because of p53 activation, we next extracted cell cycle profiles from scRNA-seq data sets. We imputed the cell cycle phase of each individual cell with the standard Seurat cell cycle scoring tool (Nestorowa *et al.* 2016). Interestingly, the *WT/G132D* mutant organoids had the highest percentage of cells in the G1 phase and the lowest percentage of G2/M cells compared to other genotypes (Fig. 3C and D). However, we did not detect any noticeable differences in cell cycle phases after irradiation in the mutant thyroid organoids, while the wild-type organoids exhibited an increase in the G1 phase population, suggesting a blockade in progression through G1 cell cycle phase (Fig. 3D). To gain more coverage of transcriptomic profile changes, we analyzed the thyroid organoids with bulk RNA-seq (bRNA-seq) to complement the scRNA-seq data using the same irradiation dosage and timing (3 Gy, 8 h). We plotted the DEG volcano plots to observe how radiation exposure affected transcriptomic patterns in the different genotypes. We observed more prominent changes in p53-regulated transcripts after irradiation in *WT/WT* and *WT/G132D* cells, while in the *WT/M134R* cells, almost no DEGs were detected (Fig. 3G, H, and I). Similarly, we detected the predicted IPA p53 pathway upregulation in *WT/WT* and *WT/G132D*, but not in *WT/M134R* (Fig. 3J, K, and L). When we inhibited p53 function, we found that only the WT/WT organoids showed a predicted activation of the p53 pathways, while no p53 downstream gene activation was detected in either of the *PTEN* mutant organoids (Supplementary Fig. 6). This has many implications in oncogenesis since a low activation of p53 in the *WT/M134R* genotype in response to irradiation can be a basis of failure to maintain genomic stability in the thyrocytes, which could contribute to tumorigenesis. We also filtered our scRNA-seq data set for only p53 transcript-positive cells and found that after irradiation, p53 signaling was predicted to be less activated in the *WT/M134R* cells while PTEN signaling was predicted to be activated in the *WT/G132D* cells (Supplementary Fig. 5). Overall, the scRNA-seq and bRNA-seq DEG and subsequent IPA consistently pointed out a divergent response upon irradiation due to the differential activation of the p53 signaling network between the two mutant genotypes.

### *PTEN* genotype-dependent p53 activation cannot be completely explained by PTEN canonical function

**Figure 4:**
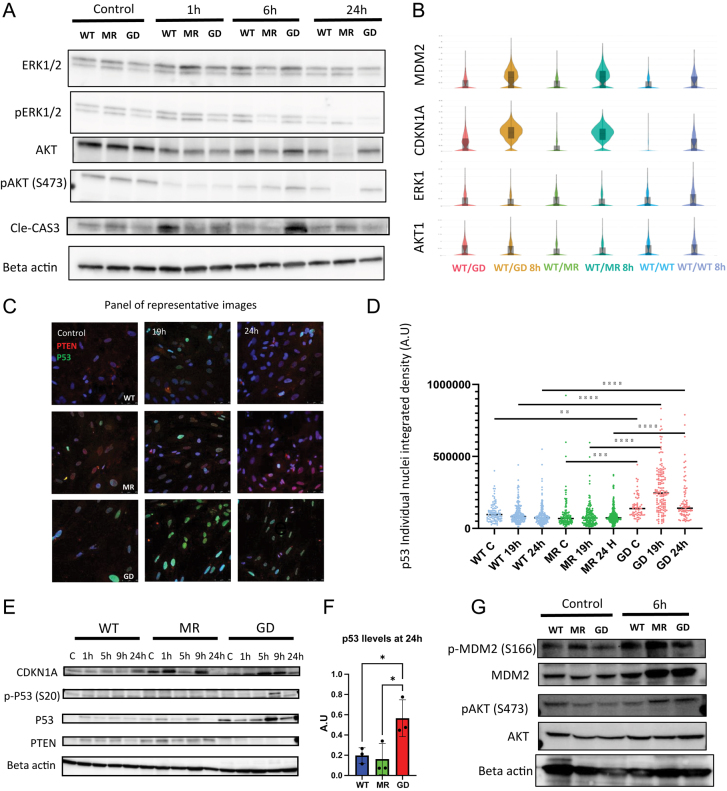
*PTEN^WT/G132D^* thyroid organoids show increased p53 protein levels. (a) Western blots of thyroid organoid lysates before and 1, 6, and 24 h after 3 Gy exposure. ERK1/2, p-ERK1/2, AKT, p-AKT(S473), and cleaved CAS3 are interrogated, and beta actin is used as a loading control. (b) Violin plot of scRNA-seq MDM2, CDKN1A, ERK1 and AKT1 transcripts in pre- and post-irradiated samples. (c) IF panel of representative images of 2D-plated thyroid organoid cells stained for PTEN and p53, before and after 19 and 24 h of 3 Gy irradiation. (d) Quantification of p53 individual nucleus-integrated density of 2D-plated thyroid organoid cells pre- and post-irradiation at 19 and 24 h. Each dot represents an individual nucleus; *F* = 19.27; *P*-value = <0.0001. (e) Western blots of thyroid organoid lysates before and 1, 5, 9, and 24 h after 3 Gy exposure. CDKN1A p-p53 (S20), p53, and PTEN are interrogated, and beta actin is used as a loading control. (f) Western blot quantification of p53 protein levels at 24 h after irradiation. (g) Western blots of thyroid organoid lysates before and 6 h after 3 Gy exposure. MDM2, p-MDM2 (S166), AKT, and p-AKT(S473) are interrogated, and beta actin is used as a loading control.

To assess which PTEN function associates with p53 activation difference among the genotypes, we analyzed the phosphorylation status of AKT and ERK1/2 as well as cleaved CASP3 levels to correlate the PTEN signaling with cell survival after irradiation. There were no obvious differences in the p-AKT (S473) levels among the three genotypes prior to irradiation (contrary to what the hiPSC western blot exhibited, Fig. 1). Upon gamma irradiation, we noticed an acute (1 h) downregulation of p-AKT (S473) followed by partial recovery (6 h). p-ERK1/2 exhibited a gradual downregulation, reaching the lowest levels at 24 h after irradiation (Fig. 4A). Overall, some variability was noted in the p-AKT/AKT levels at 24 h and in the p-ERK1/2/ERK1/2 levels at 6 and 24 h. We checked the transcriptomic status of the ERK1/2 and AKT genes and found no significant differences among the genotypes (Fig. 4B). These observations suggested that it is not the PTEN/AKT axis that regulates the predicted p53 activity patterns.

Next, we sought to evaluate the p53 protein levels and its phosphorylation status to validate the IPA p53 signaling pathway prediction. Immunofluorescence staining indicated that p53 levels were maintained at significantly higher levels in the plated thyroid cells at 0, 19 and 24 h after irradiation of *WT/G132D* (Fig. 4C and D), but not *WT/WT* nor *WT/M134R*. Next, we measured the p-Ser20 status of the p53 protein and observed an increase in protein levels accompanied by an increase in total p53 protein at 9 h post-irradiation only in *WT/G132D* and these levels remained significantly higher at 24 h after irradiation (Fig. 4E and F and Supplementary Fig. 7). Finally, we assessed the MDM2 phosphorylation status on serine 166 without observing major changes in phospho-MDM2 in the *WT/M134R* genotype organoid lysate after irradiation when compared to the other two genotypes and without showing differences in phospho-AKT among the three genotypes (Fig. 4G). Overall, we conclude that the increase in p53 protein in the *WT/G132D* cells cannot be completely explained by PTEN phosphatase activity.

### PTEN^*G132D*^ increases its colocalization and physical interaction with p53, upon irradiation, more than the PTEN^*WT*^ and *PTEN^M134R^* variants

**Figure 5:**
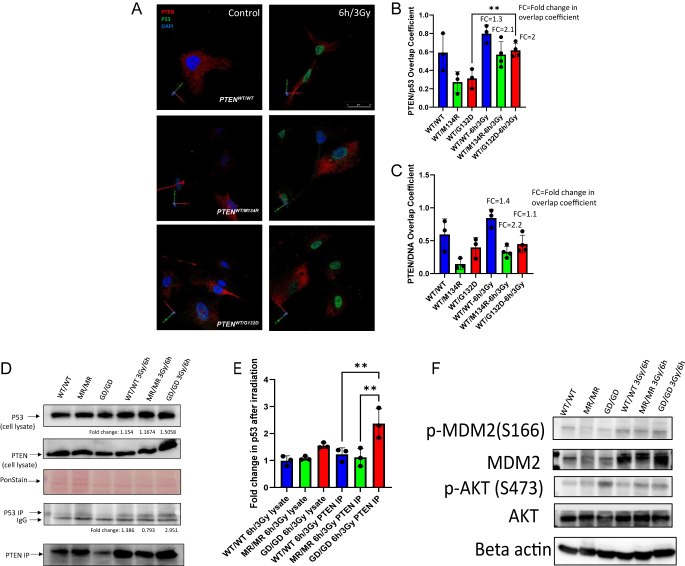
γ-irradiation increased PTEN–p53 colocalization in *PTEN^WT/G132D^* cells and the affinity between p53 and G132D PTEN. (a) IF panel of 3D reconstructions of plated thyroid organoid cells stained for PTEN and p53, before and after 6 h of 3 Gy irradiation. (b) Quantification of the overlap coefficient between PTEN and p53 before and after 6 h of 3 Gy irradiation; *F* = 2.076; *P*-value = 0.0064; FC = fold change in overlap coefficient. (c) Quantification of the overlap coefficient between PTEN and DNA before and after 6 h of 3 Gy irradiation; FC = fold change in overlap coefficient. (d) Representative images of immunoprecipitation assay of PTEN protein before and after 3 Gy irradiation in hiPSC homozygote cell lines. The upper bands are p53 and PTEN in the whole-cell lysate, the middle bands are Ponceau staining as loading control, and the lower bands are the immunoprecipitated PTEN/p53 fraction. (e) Fold change in p53 after irradiation in the total cell lysate and the PTEN/p53 immunoprecipitated fraction. (f) Western blots of hiPSCs with *PTEN^G132D/G132D^*, *PTEN^M134R/M134R^*, and *PTEN^WT/WT^* genotype lysates before and 6 h after 3 Gy exposure. MDM2, p-MDM2 (S166), AKT, and p-AKT(S473) are interrogated, and beta actin is used as a loading control.

To understand the PTEN lipid phosphatase-independent mechanism of divergent p53 activation, we interrogated the possibility of a non-canonical PTEN function to account for the increased p53 activity observed in the *WT/G132D* genotype. Since PTEN has been shown to increase p53 stability due to their direct physical interaction (Stambolic *et al.* 2001, Tang & Eng 2006), we performed a colocalization analysis pre- and post-irradiation of 2D monolayer thyroid cell cultures derived from thyroid organoids. We reconstructed 3D images of the plated thyroid organoid cells (Fig. 5A) and assessed the overlap coefficient between the PTEN and p53 signals (Fig. 5B) and the PTEN/DNA channels overlap coefficient (Fig. 5C). All genotypes showed a trend toward increased overlap coefficient after irradiation, but the only significant increase was detected in *WT/G132D* almost doubling its overlap coefficient upon irradiation. Then, we calculated the mean fold increase in the overlap coefficients for each genotype simultaneously. Overall, we observed a higher fold change increase in the PTEN/p53 overlap coefficient in the two mutants when compared to the *WT/WT* organoid cells. Using the PTEN/DNA coefficient, we only observed a trend toward more overlap of the channels after irradiation in all the genotypes (Fig. 5C). In this approach, using heterozygous cells, it was impossible to establish which *PTEN* allele is responsible for the increase in colocalization of *WT/G132D* since anti-PTEN recognizes both mutant and wild-type PTEN proteins.

To overcome this challenge, we used homozygous hiPSCs to perform immunoprecipitation of PTEN protein and tested for co-precipitated p53 in pre- and post-irradiation samples. We blotted the whole-cell lysate fraction as well as the PTEN-precipitated beads fraction (Fig. 5D). We found that upon irradiation, the p53 levels increased ∼2-fold in the PTEN pulldown, but only ∼1.3-fold in the whole-cell lysate fraction of G132D (Fig. 5D and E). We did not observe this type of p53 enrichment by PTEN immunoprecipitation in other genotypes. We then tested the PTEN downstream AKT/MDM2 phosphorylation status in the homozygote hiPSC. We found similar levels of MDM2 phospho-serine 166 in all the genotypes after irradiation. In contrast, the p-AKT levels were higher in *G132D*/*G132D* cells when compared to *M134R/M134R* and *WT/WT* cells, while there is no change in p-MDM2 (Fig. 5F). The PTEN and p53 levels were also induced by irradiation in *G132D*/*G132D* cells. Overall, these observations suggest that the induction of p53 protein levels is not due to a PTEN/pAKT-mediated mechanism but rather due to an increased physical interaction between mutant PTEN and p53.

## Discussion

The current work investigates how two *PTEN* germline variants, *M134R* (associated with DTC) and *G132D* (not associated with DTC), differentially respond to radiation-induced DNA damage in a human thyroid organoid model. The two variants exhibited a divergent DDR signal based on p53 network activity, MDM2 phosphorylation status, and PTEN–p53 physical interaction.

Analyzing the *PTEN* variant-specific DDR of thyrocytes upon irradiation would promote a fundamental knowledge base for assessing DTC risk in the individualized care of PHTS patients. We previously reported that the same *PTEN* variants used in this study differentially disrupt neurogenesis in human forebrain organoids and cell cycle checkpoints in hiPSCs (Hitomi *et al.* 2023, Kang *et al.* 2023). Interestingly, in our previous work, we found a disruption in the cadherin switch, a key molecular signature of EMT, in early neuroepithelial precursor (NEP) organoids with the *WT/G132D* genotype. However, in the current study, we detected enhanced cell migration in a wound healing assay and detachment of some cells from the main cluster of hiPSCs of *WT/G132D* genotype. These phenomena are characteristics of EMT, in which PTEN plays a crucial role (Fedorova *et al.* 2022, Akhmetkaliyev *et al.* 2023). These opposing observations may be due to the different molecular networks acting in the different tissue types (pluripotent vs NEP), which warrants further investigation. In addition, it is important to mention the differences in cell cycle between the hiPSC and the thyroid organoid. In this case, we detected more G1 phase cells in the *WT/G132D* thyroid organoids, suggesting a differentiated or senescent phenotype linked to the thyroid organoid tissue rather than the variant itself.

PTEN acts as the main regulator of PI3K/AKT signaling, which in turn induces MDM2 phosphorylation on serine 166 and 186 residues promoting its stability and translocation to the cell nuclei (Mayo & Donner 2001, Ogawara *et al.* 2002, Chibaya *et al.* 2021). This phenomenon may partially explain the higher p53 activity predicted from irradiation-induced transcriptomic profile changes and the higher p53 protein level in *WT/G132D* organoids since MDM2 serves as one of the primary modulators of the p53 activity forming a powerful feedback loop (Barak *et al.* 1993, Wu *et al.* 1993). When we looked at the PTEN/AKT/MDM2 axis in the homozygote hiPSC, we did not observe major differences in p-MDM2 (S166) between the *WT/G132D* and *WT/M134R* cells. Since the homozygous hiPSC only expresses the mutant allele, we inferred that the observations in the thyroid organoid may be due to a factor not related to the particular *PTEN* mutation, but rather due to other factors such as heterodimerization and allele dosage (Lee *et al.* 2018).

Finally, when assessing the non-canonical activity of PTEN in relationship to p53, we discovered that the PTEN–p53 physical interaction is notably induced in the *G132D/G132D* genotype when compared to the *M134R/M132R* and *WT/WT* hiPSCs. This interaction has been reported to stabilize p53 by preventing MDM2-mediated degradation (Stambolic *et al.* 2001, Tang & Eng 2006, Chang *et al.* 2008). In summary, the G132D hiPSC could be acting through phosphatase-dependent and phosphatase-independent means to upregulate p53, while the M134R hiPSC could not in γ-irradiated thyroid organoids. Previous work has shown that double hits to PTEN are fundamental drivers of DTC in PHTS (Plitt *et al.* 2024). We believe that our homozygote system could potentially represent this phenomenon.

This phenomenon may have clinical implication since the *M134R* allele, but not *G132D*, has been previously associated with the development of DTC in our PHTS patient cohort. PTEN and p53 play a pivotal role in regulating DNA repair, cell cycle arrest, senescence, and apoptosis (Levine 2020). The failure to activate the p53 pathway to high levels may promote genomic instability, making thyroid cells with the *WT/M134R* genotype more prone to tumorigenesis after exposure to genotoxic ionizing radiation than those with the *WT/G132D* genotype. The current study demonstrates the utility of iPSC-derived organoids with specific gene editing as a model to enable individualized medicine for pleiotropic hereditary diseases such as PHTS.

As a limitation of our work, we acknowledge that due to the limited number of clones in each genotype, we cannot rule out a clonal effect in the phenomena described here.

## Supplementary materials



## Declaration of interest

The authors declare that there is no conflict of interest that could be perceived as prejudicing the impartiality of this work.

## Funding

The work was funded, in part, by the Ambose Monell PTEN Switch Grant (to CE), the Lisa Dean Mosley Foundaiton Grant (to CE and MH), and the Cleveland Clinic Caregiver Catalyst Grant (to CE and JAV).
