# Supplementary material for: Divergent PTEN–p53 interaction upon DNA damage in a human thyroid organoid model with germline PTEN mutations
Source: Endocr Relat Cancer. 2025 Mar 8;32(4):e240216. doi: 10.1530/ERC-24-0216 (PMC11906015; doi:10.1530/ERC-24-0216)
Supplement: Supplementary file 1 [file supplementary_materials.pdf]

Supplemental Fig. 1

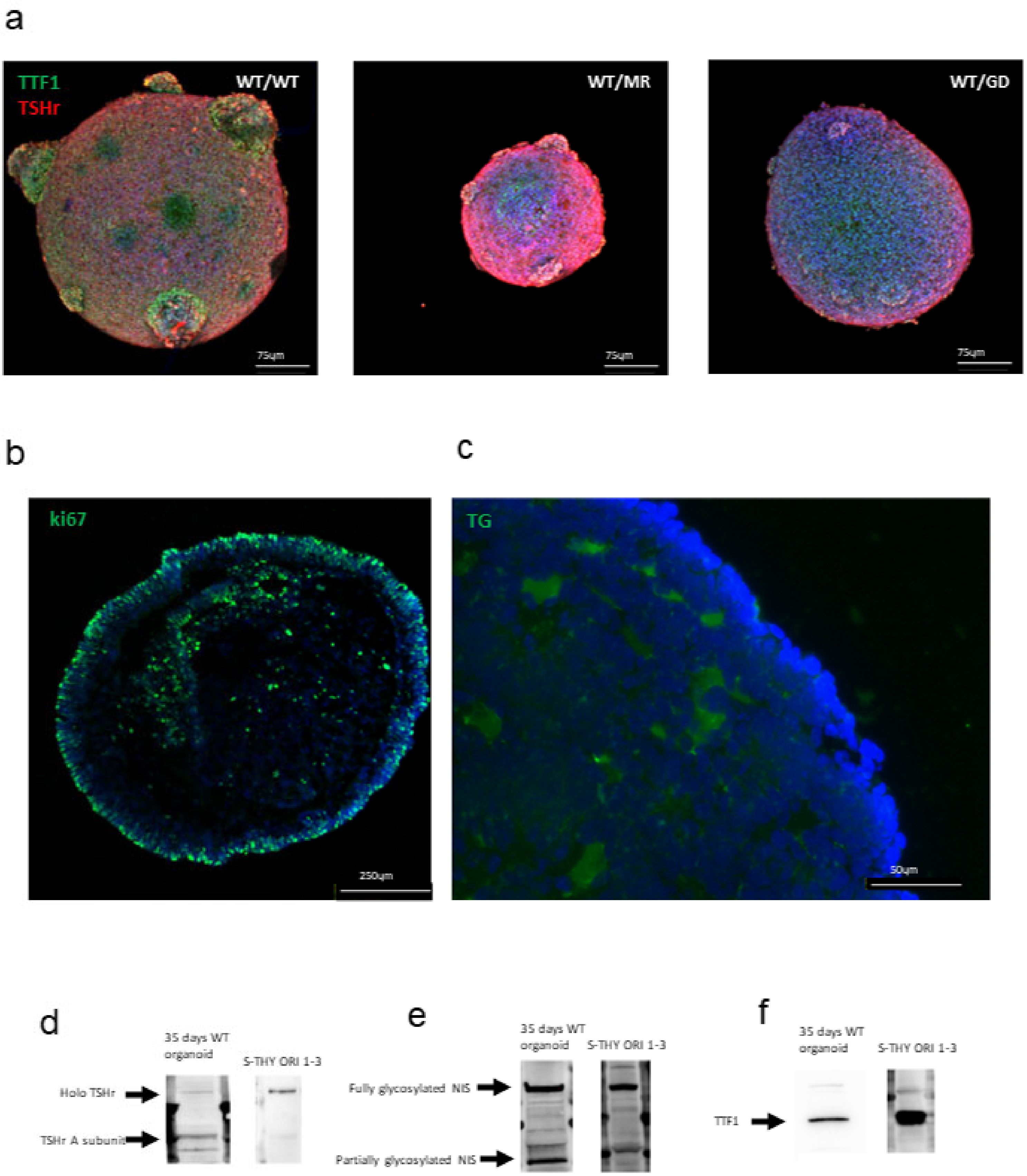

Supplement Figure 1. 3D reconstruction of human thyroid organoids and thyroid specific protein expression (a) *PTEN* (WT/WT), (WT/MR), and (WT/GD) z-stack projections of human thyroid organoids. (b) Proliferating cells (ki67+) of human thyroid organoid (c) intrafollicular staining of thyroglobulin in thyroid organoid (d) Western blot of WT day 35 thyroid organoid lysate for TSHr (e) Western blot of WT day 35 thyroid organoid lysate for NIS (f) Western blot of WT day 35 thyroid organoid lysate for TTF1. STHY-ORI 1-3 serves as positive control.

Supplemental Fig. 2

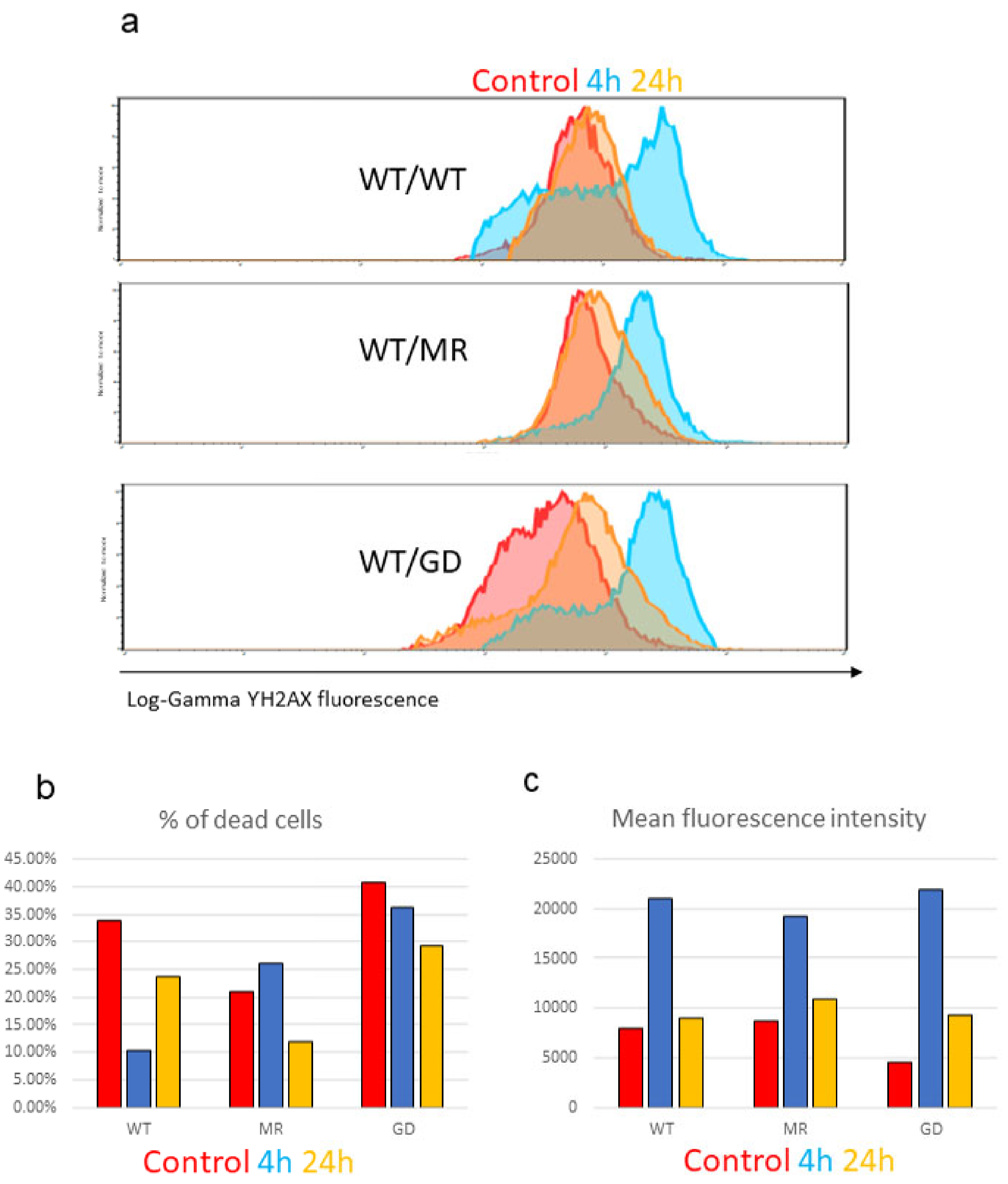

Supplement Figure 3. **FACS experiments confirm the presence of DNA damage after 3Gy exposure in thyroid organoid cells** (a) *PTEN* (WT/WT), (WT/MR), and (WT/GD) histogram of gamma H2AX fluorescence at 0h (control), 4h and 24h after 3Gy dose. (b) Percent of dead cells in each genotype before, 4h and 24h after irradiation (d) Mean fluorescence intensity in each genotype before, 4h and 24h after irradiation

Supplemental Fig. 3

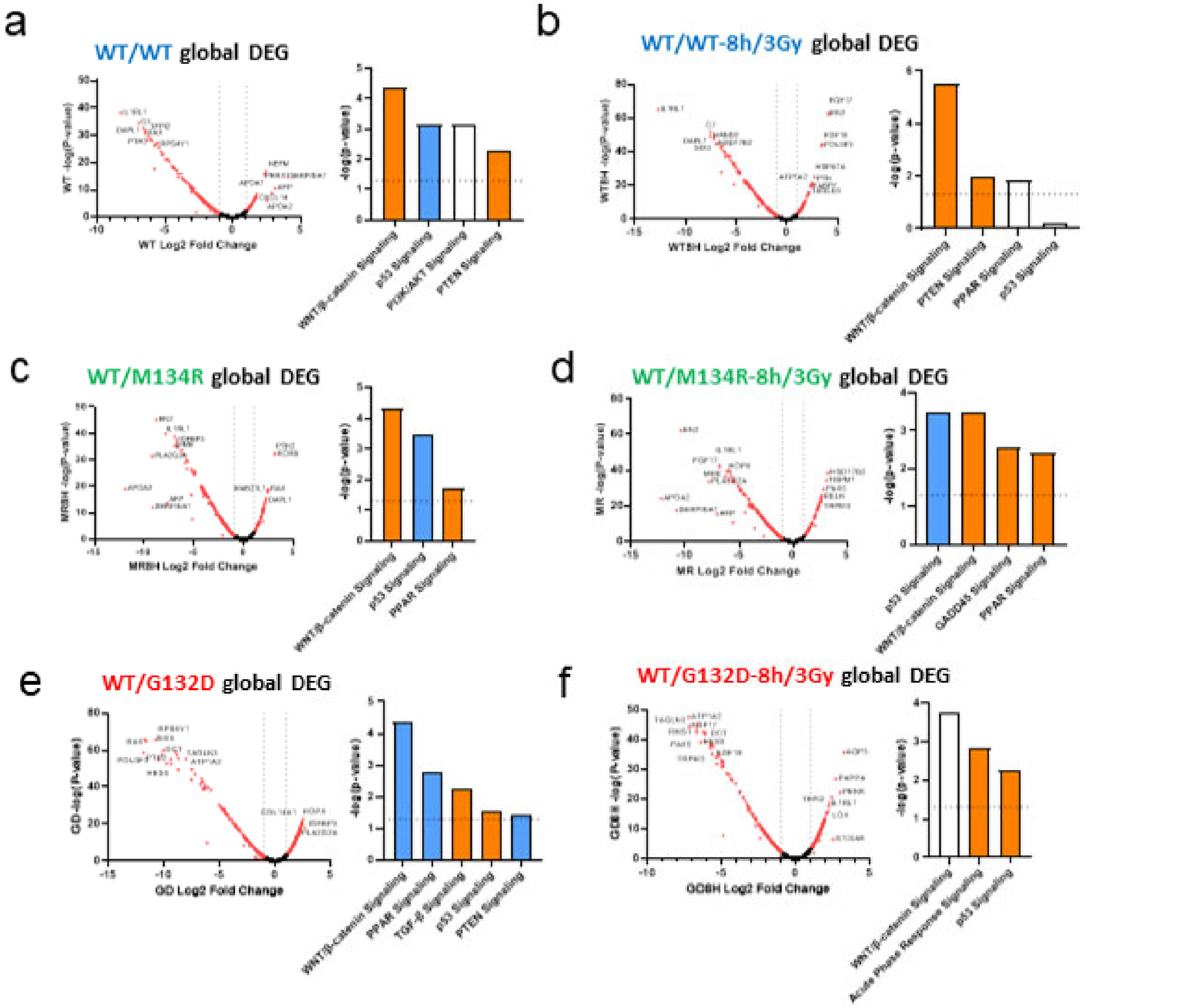

Supplement Figure 3. scRNAseq data set global comparison retain the p53 activity predictions (a) IPA predictions of up/down regulated pathways in WT/WT global comparison. (b) IPA predictions of up/down regulated pathways in WT/WT8h/3Gy global comparison. (c) IPA predictions of up/down regulated pathways in WT/MR global comparison. (d) IPA predictions of up/down regulated pathways in WT/MR8h/3Gy global comparison. (e) IPA predictions of up/down regulated pathways in WT/GD global comparison. (f) IPA predictions of up/down regulated pathways in WT/GD8h/3Gy global comparison.

Supplemental Fig. 4

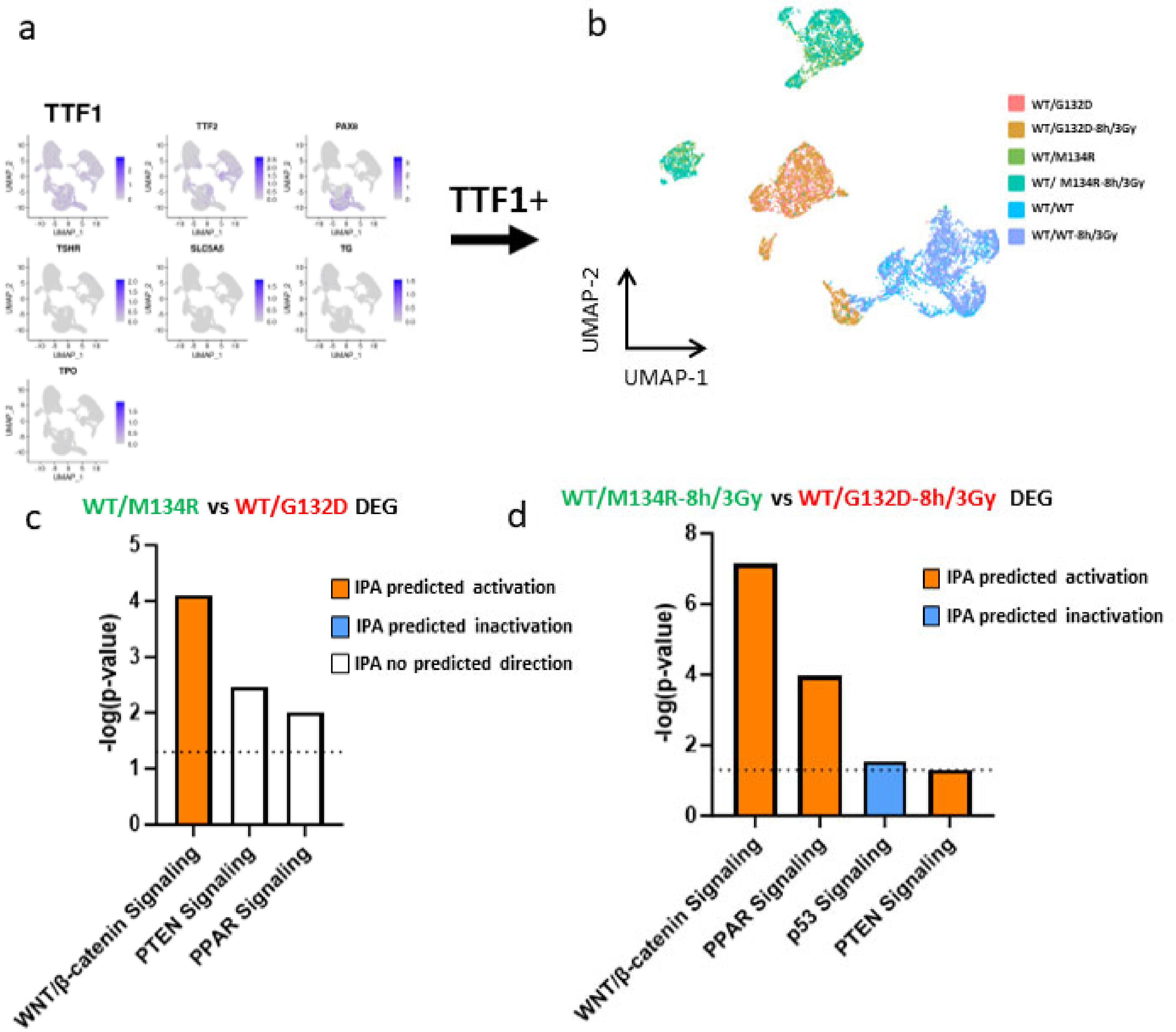

Supplement Figure 4. scRNAseq data set filtration by thyroid identity genes retain the p53 activity predictions (a) UMAPs of thyroid specific genes (b) UMAP of TTF1+ beads colored by the different sample types (c) IPA predictions of up/down regulated pathways in WT/MR vs WT/GD (e) IPA predictions of up/down regulated pathways in WT/MR vs WT/GD

Supplemental Fig. 5

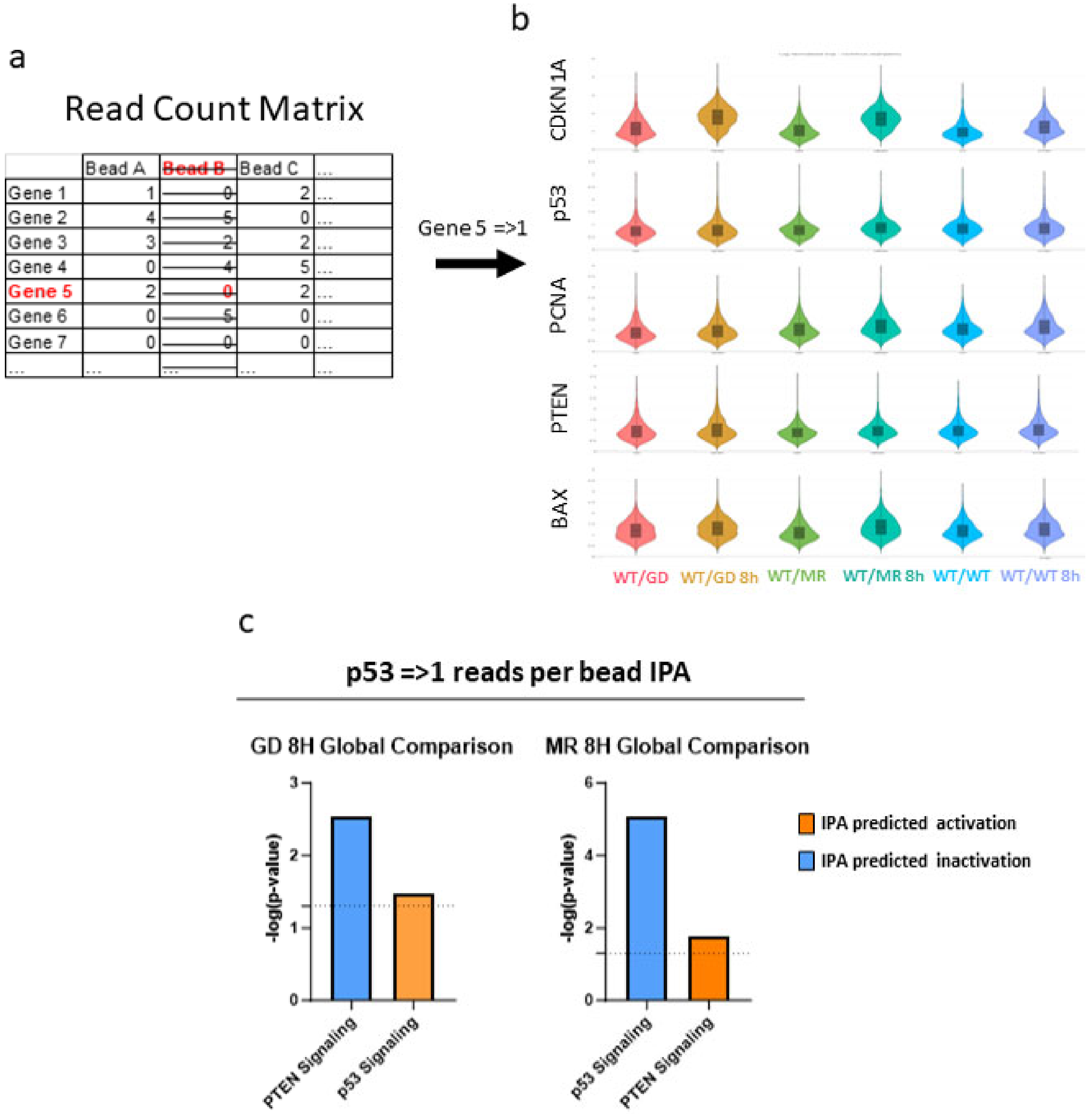

Supplement Figure 5. scRNAseq data set filtration DDR genes retain the p53 activity predictions (a) example on how we filtrated the beads with 0 reads for a specific gene (b) Violin plots DDR specific transcrips after 0 read filter (c) IPA predictions of up/down regulated pathways in WT/MR8h/3Gy and WT/GD8h/3Gy global comparison.

Supplemental Fig. 6

a

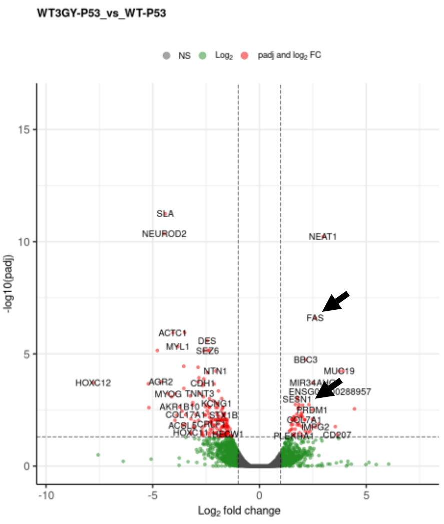

b

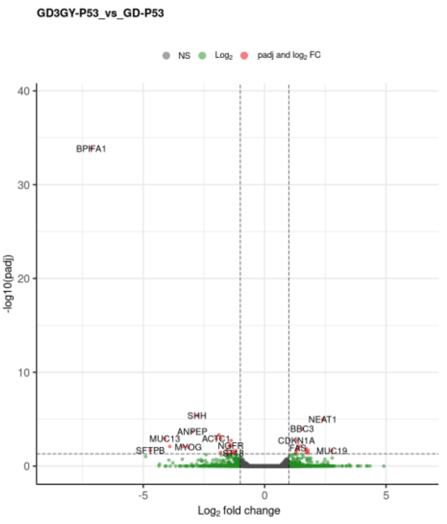

c

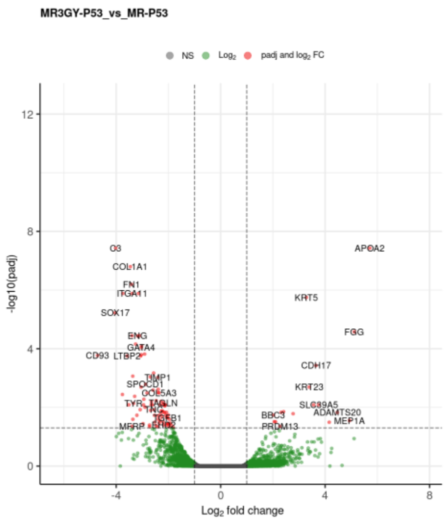

d

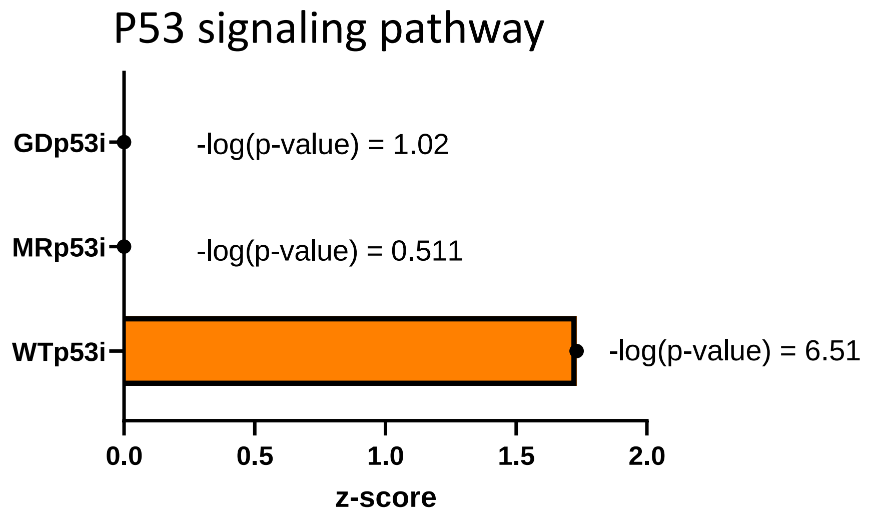

Supplement Figure 6. scRNAseq data set global comparison retain the p53 activity predictions (a) Volcano plot up/down regulated transcripts in WT/WT8h/3Gy vs WT/WT organoids. (b) Volcano plot up/down regulated transcripts in WT/MR8h/3Gy vs WT/MR organoids (c) Volcano plot up/down regulated transcripts in WT/GD8h/3Gy vs WT/GD O Gy organoids (d) IPA results of the p53 signaling pathway in each genotype.

Supplemental Fig. 7

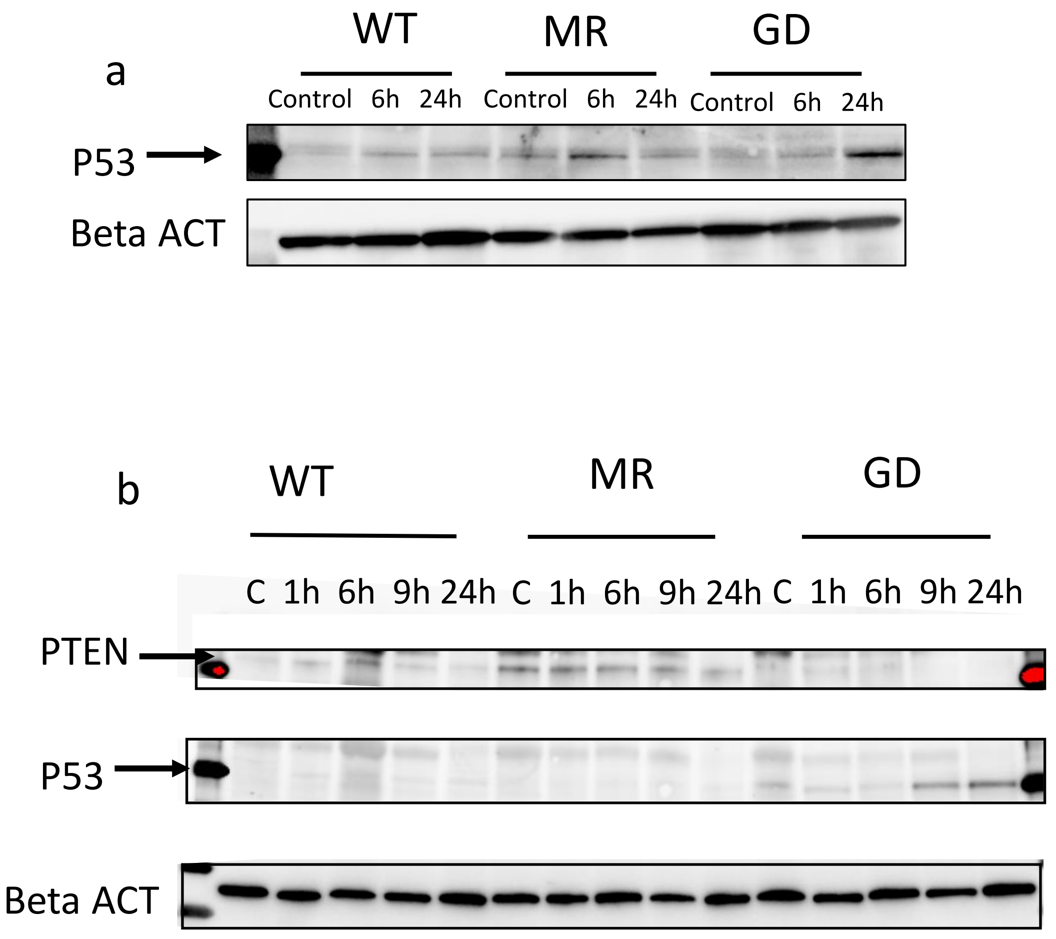

Supplement Figure 7. p53 protein levels in two independent experiments (a) Western Blot of p53 after irradiation exposure in the three genotypes at 6h and 14h . (b) Western Blot of p53 and PTEN after irradiation exposure in the three genotypes at 1h, 6h, 9h and 24h.

Supplemental Table 1

Supplement Table 1: Primary and secondary antibodies used

Antibodies	Cat. no.	Vendor
SOX2	MAB2018	R&D systems, Minneapolis, MN
PAX9	ab28538	Abcam
AFP	SAB3500533	MilliporeSigma
TTF-1	ab76013	Abcam
Cas-3	9661S	Cell Signaling Technology
TPO	PA5-81070	Invitrogen
PAX-8	ab191870	Abcam
PTEN	ABM-2052	Cascade Bioscience Inc., Akron, OH
TSHr	ab123488	Abcam
TG	ab168344	Abcam
AKT	9272	Cell Signaling Technology
pAKT S473 (D9E)	4060	Cell Signaling Technology
p44/42 MAP Kinase (Total)	9102	Cell Signaling
P-p44/42 MAPK(T202/Y204) (20G11)	4376	Cell Signaling
p53	2527	Cell Signaling
p-S20 p53	9287S	Cell Signaling
MDM2	ab259265	Abcam
P-S166 MDM2	ab131355	Abcam
β ACTIN	AM4302	Invitrogen ThermoFisher Scientific
p21	sc-397	SantaCruz
ALEXA FLUOR® 488, 594	A11006, A11008, A32740	Invitrogen ThermoFisher Scientific
Horse radish peroxides anti-Mouse, Rabbit IgG(H+L)	PRW4021, 4011	Promega, Madison, WI
Mouse Anti-rabbit IgG (Light-Chain Specific)	93725	Cell Signaling
Mouse Anti-mouse IgG (Light-Chain Specific)	58802S	Cell Signaling
PE anti-H2A.X Phospho (Ser139) Antibody	613411	Biolegend
